# Designing a Web-Based Psychological Intervention for Patients With Myocardial Infarction With Nonobstructive Coronary Arteries: User-Centered Design Approach

**DOI:** 10.2196/19066

**Published:** 2020-09-17

**Authors:** Sophia Monica Humphries, Elisabet Rondung, Fredrika Norlund, Örjan Sundin, Per Tornvall, Claes Held, Jonas Spaak, Patrik Lyngå, Erik M G Olsson

**Affiliations:** 1 Department of Women's and Children's Health Uppsala University Uppsala Sweden; 2 Department of Psychology and Social Work Mid Sweden University Östersund Sweden; 3 Department of Clinical Science and Education Karolinska Institute Stockholm Sweden; 4 Department of Medical Sciences, Cardiology Uppsala Clinical Research Center Uppsala University Uppsala Sweden; 5 Department of Clinical Sciences Danderyd Hospital Karolinska Institute Stockholm Sweden

**Keywords:** web-based intervention, iCBT, myocardial infarction, nonobstructive coronary arteries, patient involvement, psychological treatment, MINOCA, takotsubo cardiomyopathy

## Abstract

**Background:**

The involvement of patient research partners (PRPs) in research aims to safeguard the needs of patient groups and produce new interventions that are developed based on patient input. Myocardial infarction with nonobstructive coronary arteries (MINOCA), unlike acute myocardial infarction (MI) with obstructive coronary arteries, is presented with no significant obstructive coronary artery disease. Patients with this diagnosis are a subset of those diagnosed with traditional MI and often need more psychological support, something that is presently not established in the current treatment scheme in Swedish health care or elsewhere, to our knowledge. An internet-delivered intervention might offer patients with MINOCA the opportunity to access a psychological treatment that is tailored to their specific needs after MINOCA and could therefore supplement the existing medical care in an easily accessible format.

**Objective:**

This paper aims to describe the development of a therapist-guided, internet-delivered psychological intervention designed specifically for patients with MINOCA.

**Methods:**

The study used a participatory design that involved 7 PRPs diagnosed with MINOCA who collaborated with a team consisting of researchers, cardiologists, and psychologists. Intervention content was developed iteratively and presented to the PRPs across several prototypes, each continually adjusted and redesigned according to the feedback received. The intervention and experience of it were discussed by PRPs in a final meeting and then presented to a panel of 2 clinical psychologists and a cardiologist for further input.

**Results:**

The outcome of the collaboration between PRPs and the research group produced a web-based psychological 9-step program focusing on stress, worry, and valued action. The input from PRPs contributed substantially to the therapy content, homework tasks, interactive activities, multimedia, and design presentation.

**Conclusions:**

Working with PRPs to develop an intervention for people with MINOCA produced a web-based intervention that can be further evaluated with the goal of offering a new psychological treatment option to a patient group currently without one. Direct contribution from PRPs enabled us to obtain relevant, insightful, and valuable feedback that was put towards the overall design and content of the intervention.

## Introduction

### Background

Myocardial infarction with nonobstructive coronary arteries (MINOCA) comprises a subset of patients with myocardial infarction (MI) [1-3]. Depending on the defining criteria of nonobstructed arteries, prevalence lies somewhere between 1% and 14% of all patients with MI [4-6]. Around 50% of these patients are further classified as having takotsubo cardiomyopathy (TTC) and therefore constitute a large proportion of the literature regarding patients with MINOCA. MINOCA is briefly defined as an acute MI that presents on coronary angiography with no significant obstructive coronary artery disease (CAD). The distinction between TTC and other types of MINOCA can be challenging, but TTC is a condition more distinctly characterized by a sudden temporary weakening of the muscular portion of the heart. The remaining patients are often grouped into various subcategories, such as plaque and nonplaque mechanisms, or even “undefined” [7]. A detailed description of diagnostic procedures was recently published [4].

Diagnosis and prognosis separate patients with MINOCA from patients with MI due to obstructive CAD [8], as do patient characteristics showing that MINOCA occurs predominantly in women and at a younger age than MI due to obstructive CAD. Around 30% of all patients with MINOCA have had a previous diagnosis of psychiatric illness [9], and more than half of patients with MINOCA report some kind of emotional or physical stressor prior to the hospital admission compared with matched patients with CAD, who report a stressor in less than 15% of cases [10]. The risk of experiencing psychological distress, such as symptoms of anxiety or depression, after MI due to obstructive CAD is already reportedly quite high compared with controls [11], and patients with MINOCA report levels even higher [10].

For patients who have experienced MINOCA, the time following the acute event can be just as daunting and stressful as the event itself. Many report a low quality of life owing to this aftermath [12,13], and this coupled with the unclear etiology of the diagnosis can make the time following the acute event increasingly open to anxiety and depression. Qualitative interviews with patients with TTC have explored the experience of living with these problems, as well as the impact on long-term stress. A major finding has been that most, if not all, have described their lives as being lived under constantly stressful circumstances [14] and that they feel limited in their lives regarding future health or long-term recovery [15]. A new study into stress associated with TTC reported that more than half of the patients in the study were still on part-time sick leave 6 months post-onset of the diagnosis [16]. Patients also experienced more vulnerability to stress after the event, which consequently led to self-reported sleep disturbances, memory loss, and difficulties concentrating. Thus, the implications of MINOCA appear to encompass a lot more than the acute event itself.

Patients in this diagnostic group have reported that it can be challenging to comprehend having a diagnosis of MI, as well as being unfamiliar with the term MINOCA, or MI with “normal” arteries [15]. Health care staff often give vague answers or explanations of the cause, which in turn may trigger more anxiety [17]. The insufficient knowledge surrounding a diagnosis of TTC or MINOCA is a challenge in cardiac rehabilitation. Finding ways to manage the stress and psychological impact of the event, in addition to addressing any pre-existing problems that might have been present, are also important factors that should be targeted in cardiac rehabilitation for patients after MINOCA [9].

Cognitive behavioral therapy (CBT) is a longstanding and supporting therapy for treatment of mood and anxiety disorders, among many others. Guidelines from the National Institute for Health and Care Excellence (the so-called NICE guidelines) recommend CBT in the referral advice and treatment for subthreshold symptoms and mild to moderate common mental health disorders [18]. Support for using face-to-face CBT to treat symptoms of anxiety and depression already holds some benefits when compared with standard care, both in patients discharged after a coronary event [19] and with other somatic illnesses, such as chronic obstructive pulmonary disease [20]. According to a meta-analysis, psychological treatments produce lower mortality rates and reoccurrences in cardiac patients, although this effect has mainly been demonstrated in men [21]. CBT has successfully been converted to be provided online in the treatment of many mental health disorders. Studies have shown that using internet-based CBT (iCBT) to treat cases of mild anxiety and depression are just as effective as face-to-face therapy and hold many benefits over using traditional forms of CBT [22]. For those without easy access to mental health treatment, iCBT is an acceptable mode of treatment, even at long-term follow-up [23]. In the case of diverse groups, such as cardiac patients and stress-related disorders, iCBT appears to be better at lowering stress-related outcomes than its active or waitlist control counterparts [24-26]. With regard to patient acceptability, a recent meta-analysis showed that iCBT for the treatment of depressive and anxiety disorders is highly effective in clinical practice and that patients reported high levels of satisfaction [27]. On the contrary, some studies have reported that patient acceptability of iCBT is lower when compared with face-to-face psychological therapy [28,29]. However, none have explored this in patients with a MINOCA diagnosis. Since only a handful of small studies have explored patient acceptability of iCBT in a patient population experiencing cardiac-related illness, there is arguably an increasing need for such initiatives [30,31]. Many studies also lack qualitative methods and detailed methodological reporting, as well as investigation of patient opinions of online therapy. This is especially the case for treatment of anxiety and depression among those with cardiac-related illnesses [32]. The lack of existing and conclusive research findings into the psychological effects of MINOCA means that intervention development could benefit from involving patients in the process. Patient and public involvement (PPI) in the development of a new intervention is based on the argument that it brings out a product that is relevant to the population [33] and gathers new insights [34], which in turn should help to map potential problems before they arise in a trial setting.

There are different ways to include patients in the process of the early development of an intervention study. According to guidelines, when the patient group is underserved or the intervention is novel, particularly for a unique patient group, it is of great importance to involve patients directly by using a patient-centered approach in both the intervention and the study development [35]. Using PPI to improve future internet-based intervention studies involves patients in the development stage of the intervention rather than just in the piloting and feasibility stages and beyond [35]. The current literature on patients with MINOCA is fairly limited, particularly with regard to their psychological needs, and we know of no psychological treatment specific to patients with MINOCA. In the process of applying a user-centered design, we also aim to lower the likelihood of attrition to the final intervention with a program that is better tailored to the needs of patients with MINOCA. Therefore, the uniqueness of the patient group, the limited knowledge about their needs, and the nonexistent psychological treatment options are together strong arguments supporting the development and testing of an iCBT treatment through PPI methods.

### Aims and Objectives

One aim of this paper is to outline how PPI has contributed to the development of an internet-based intervention for treating symptoms of psychological distress experienced by patients after MINOCA. In this sense, we argue for why it is important to work *with*, rather than just *for*, patients with MINOCA to avoid making assumptions about the intervention design or without justifiable inclusion of certain methods and content.

Another aim is to present the procedure in which we designed and built the intervention by sharing the development process and strategies used throughout. This includes how we used patient feedback throughout each step of the intervention, the suggestions for changes during developing the intervention, and how those modifications were incorporated into the intervention (or considered for future improvement). Challenges will also be discussed in relation to developing a complex web-based intervention with collaboration from patients.

## Methods

### Patient Participatory Methods and Recruitment

Patients were involved at the collaboration level described in PPI protocols [36], reflecting their ongoing and shared level of contribution to the intervention development, and they are referred to as patient research partners (PRPs). PRPs were considered active members of the wider research group; they were involved throughout the entirety of the development and participated in consultation and testing of developed material.

This study recruited participants from Södersjukhuset Hospital in Stockholm using convenience sampling methods. The study initially began in May 2018 with 3 PRPs who took part in the first focus group discussion. The remaining PRPs were recruited gradually over a 6- to 8-month period from the study start, leading to a total of 7 PRPs, although attendance of in-person meetings varied depending on participant availability for the given date and time.

### Participants

A total of 7 adults (5 female) participated, all with a diagnosis of MINOCA within 5 years prior to the start of the study and who lived near or were able to commute to Stockholm and were able to read and understand Swedish. Of the 7 PRPs participating in the study, 2 had confirmed TTC and the remaining had unspecified diagnoses of MINOCA. The median age of the group was 58 years and the time since first diagnosis to study inclusion ranged from 2 weeks to 5 years. Informed consent was obtained prior to the formal development process. All agreed to participate voluntarily, to speak about their experience and the psychological impact of MINOCA on their health, and to actively test the intervention as it was developed while providing feedback and insight. Ethics approval was granted by the Regional Ethical Review Board in Stockholm (Dnr 2018/1434-31/1 and 2018/2406). Approval was obtained for the use of the image in this paper by the participants.

### Project Management

The research team consisted of 4 clinical psychologists (coauthors ER, FN, ÖS, and EMGO), 3 cardiologists (coauthors CH, PT, and JS), 1 cardiac nurse (PL), and 1 PhD student with a background in psychology (SMH). The clinical background of the group was thus within the fields of psychology and cardiology. In addition to these members, web developers were also involved in the digitalization process and in implementing design features on the web platform. The research team decided on a set of requirements to guide the development a priori and kept these in focus throughout the development (see Textbox 1). These criteria were mainly for the team’s own use and have not been formally evaluated but will be reflected in the planned feasibility and randomized controlled trial (ClinicalTrials.gov NCT04178434). The core research team worked according to a schedule decided at the start of the development process and the intervention was finalized towards the end of 2019. Throughout this process, web-based development group meetings took place weekly and included the research members involved in creating the intervention. These often included discussions over content, exercises, and visual components. The creation of the digital intervention content began January 2019 and was split across 5 prototype phases, originally planned as 4 but extended due to time constraints and participant feedback. All written components of the treatment were initially created in Word documents (Microsoft Corp) and then transferred to be accessed via the Uppsala University Psychosocial Care Programme (U-CARE) portal, an online platform that enables delivery of psychological interventions over the web (used, for example, in the U-CARE heart study [37]). A more detailed description of the process is described in the upcoming sections*.*

**A priori intervention requirements.**

1. The intervention should be intended to suit the majority of patients with myocardial infarction with nonobstructive coronary arteries (MINOCA) who have psychological distress.
2. The intervention should aim at reducing the psychological suffering that is common among these patients.
3. The intervention should be accessible, preferably an eHealth intervention.
4. The intervention should, at least to some extent, be flexible and possible to adopt to new technological developments.
5. After being developed, the intervention should be a low-cost intervention (less costly than a face-to-face intervention).
6. The intervention should be possible to implement and available to most patients with MINOCA in Sweden early after the cardiac event (implementation is not part of the project, but implementation should be kept in mind).
7. The intervention should not be longer than 3 months, preferably shorter.
8. The intervention should be possible to evaluate in randomized trials.
9. The intervention should be mapped or described in a detailed way, making it possible to replicate. (This means that all interventions should be linked to a behavior change mechanism that in turn is related to desired outcomes.)
10. The intervention should be developed in collaboration with potential end users (ie, patients with MINOCA).

Based on several focus group discussions (FGDs) with patients with MINOCA, scoping searches of the literature, and input from members of the core study group with experience working with patients with MINOCA or patients with associated symptomatology of mental disorders, the treatment targets were established. These helped inform the basis of the intervention, in combination with the prespecified requirements previously mentioned.

The early-stage FGDs focused on personal experiences and in turn provided in-depth material for a needs assessment, something that is recommended as the first step when designing an intervention, according to intervention mapping approaches [38]. This needs assessment worked to analyze the problems to identify what areas of change the intervention should target in patients with MINOCA. Furthermore, with the strong evidence base for CBT in mind, it was deemed appropriate to apply the fundamental and theoretical model of CBT for treating stress and anxiety in patients with MINOCA.

### Intervention Development Procedure

The research group worked from a development plan that used a stepwise approach throughout the continued development of the intervention. The process of involving PRPs and prototyping took inspiration from the concept of user-centered design (UCD). The UCD approach views end users as pivotal to the design process of product development and is one way to avoid a one-directional relationship between the developers and the product, which often occurs when creating evidence-based treatments without end user involvement in the design process [39].

### Early-Stage Focus Group Discussions

The first instances of PRP involvement included several of the PRPs who met with 3 of the researchers (PL, ER, and FN) to discuss their experiences of MINOCA in a broad manner. A total of 4 group-style discussions took place during a period of 6 months, and the number of PRPs attending each discussion varied depending on their availability and the number of participants recruited at that point in the study. The idea of these discussions was to gather as much information as possible about (1) the problems or psychological suffering encountered as a result of the MINOCA; (2) the type of help patients would like to receive if offered to them in a digital format; (3) the format, length, features, and possible supplementary material best suited for the intervention; and (4) their opinions on relevant outcomes and specific psychological evaluation questionnaires.

### Development and Presentation of Material

Following the FGDs, the process of building the intervention began with creating the treatment content. This was done by the psychologists (coauthors ER and FN) and was developed over a period of roughly 4 to 6 weeks per prototype. Content was then uploaded online and added to a demo study in the U-CARE portal. In the initial stage, PRPs were given user log-ins that enabled them to access the same account throughout the testing. A period of approximately 10 to 14 days was allotted to the PRPs to work through the newly developed content during each testing stage. The idea was to allow enough time to actually complete the homework tasks that accompanied the content, read through the text, and look through the media content. Navigating the portal website and sending instant messages were also features the PRPs were encouraged to test. Shortly after working through the material and testing the features of the portal, PRPs were contacted by the psychologist assigned to them (ER or FN). Brief one-on-one unstructured telephone interviews were conducted that focused on PRPs’ feedback on the language, content, exercises, and experience of the portal, as well as anything else they could think of. At times it was difficult for the participants to be specific enough to advise concrete changes. This was expected, and researchers did their best to ask follow-up questions. The team then used this feedback to rebuild and redesign content, features, and other requirements for the specific content in question. This process was repeated for all stages of the intervention until completion. See Table 1 for an overview. This overall approach made the process an iterative one, as the constant redesign and evaluation of the intervention allowed for adjustments and continuous improvements before a final treatment manual was established.

**Table 1:** Process stages of the development of the intervention.

Step	Type	Content	Exercises	Attendees
Panel discussion	FGD^a^ with patient research partners group	Open discussion, information gathering	None	2 researchers, 3 patient representatives
Panel discussion	FGD with patient research partners group	Open discussion, identification of problems	Asked to go through some questionnaires, including the CAQ^b^	3 researchers, 3 patient representatives
Panel discussion	FGD with patient research partners group	Open discussion, thoughts and feedback of material, and ideas for iCBT^c^ intervention	Were presented with a brochure	3 researchers, 3 patient representatives
Panel discussion	FGD with patient participatory group	Open discussion, some testing of relevant material, feedback used to gauge usefulness	Reviewed material from an existing online intervention, tested a homework exercise on fear after a cardiac event, reviewed a video interview with a cardiologist talking about MINOCA^d^	2 researchers, 2 patient representatives
Portal introduction	Phone call with patient representatives	Instructions/introduction to online portal	Logging in to the portal, testing of user account	N/A^e^
Prototype 1	Online iCBT testing	Introduction and “Fear after MINOCA” content	Describe own experience of MINOCA	7 patient representatives invited, of which 6 logged in
Feedback	Telephone feedback interviews	Feedback via phone	N/A	6 patient representatives
Prototype 2	Online iCBT testing	Stressors and stress reactions	Listing own stressors and self-observation of stress reactions	6 patient representatives
Feedback	Telephone/mail feedback interviews	Feedback via phone	N/A	6 patient representatives
Prototype 3	Online iCBT testing	The importance of consequences, recovery, and relaxation	Trying alternative behaviors and relaxation exercises	6 patient representatives
Feedback	Telephone feedback interviews	Feedback via phone	N/A	6 patient representatives
Prototype 4	Online iCBT testing	Values	Formulating values and planning activities accordingly	6 patient representatives
Feedback	Telephone feedback interviews	Feedback via phone	N/A	6 patient representatives
Prototype 5	Online iCBT testing	Summary, evolution, and maintenance	Formulating a plan for relapse prevention and future development	4 patient representatives
PRP^f^ panel meeting and interviews	Open-group interview with semistructured guide	Overall feedback on the intervention and the process of being part of a patient panel	N/A	2 researchers, 4 patient representatives
Expert panel meeting	Discussion of the intervention from the perspectives of psychologists and cardiologists with previous knowledge/experience	Current version of the intervention with all 9 steps and material	Going through content in own time, online and through the PDF handout version	2 researchers, 2 external psychologists, and 1 external cardiologist

^a^FGD: focus group discussion.

^b^CAQ: Cardiac Anxiety Questionnaire.

^C^iCBT: internet-based cognitive behavioral therapy.

^d^MINOCA: myocardial infarction with nonobstructive coronary arteries.

^e^N/A: not applicable.

^f^PRP: patient research partner.

### Final Panel Meeting With PRPs

Two of the researchers (EMGO and SMH) interviewed PRPs with the purpose of gaining insight on not only the final intervention (content, exercises, aesthetics, among others) but also the experience of being involved in the process as well as any extra remarks that may have otherwise been missed. The translated interview guide can be found in Multimedia Appendix 1. This was followed by a dinner gathering with the whole research group and patient research partners together, allowing for discussion to continue more informally.

### Expert Panel Meeting

The final step in the development of the intervention included input from 3 experts who made up what we refer to as the expert panel. This consisted of external-only members, 2 psychologists and 1 cardiologist, and were consulted with the purpose of providing feedback from the perspectives of professionals working within their respective fields. The expert panel were sent the latest version of the full intervention manual in a written PDF format and provided with secure log-ins to the U-CARE portal as test users 1 week before the physical meeting. The panel met with 2 of the researchers from the core group (EMGO and SMH) to provide their feedback and comments on the intervention and content from the perspectives of the clinical psychology and cardiology fields.

## Results

### Final Intervention Design

**Figure 1:**
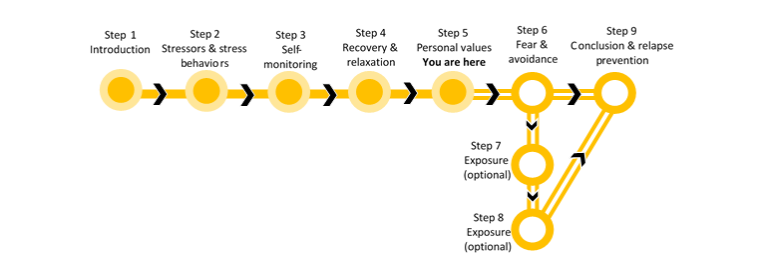
An overview of the steps in the intervention.

The final intervention design resulted in a 9-step online psychological treatment for patients with MINOCA. These are outlined as (1) introduction, (2) stressors and stress behaviors, (3) stress-specific self-monitoring, (4) recovery and relaxation, (5) personal values, (6) fear and avoidance, (7) exposure, (8) exposure continued, and (9) conclusions and relapse prevention. The work-through rate was aimed at participants completing an average of 1 step per week, resulting in an estimated 2 to 3 hours of work per week, based on reports from the PRPs. Figure 1 shows the brief overview of the intervention as presented to the participants during treatment.

The first step includes an introduction, with an overview of the intervention and several video interviews. These were made specifically for the intervention to provide information that was lacking in the current care, as previously mentioned in the FGDs. These interviews focus on the physiology of MINOCA from a cardiologist’s standpoint, a nurse talking about the acute phase of patient care, psychological reactions after MINOCA, and what it is like to experience MINOCA from a patient perspective. Stress was a commonly occurring topic in the FGDs, as well as being a well-known problem experienced by patients with MINOCA in the literature. Therefore, the next 2 steps cover stress-focused content and related exercises. Step 2 mainly focuses on psychoeducational aspects and on how to monitor stressors and stress situations in different contexts. Step 3 looks at how one can identify stress and its consequences. In Step 4, the importance of recovery and relaxation is introduced, including a small focus on sleep behavior. Step 5 encourages participants to focus on personal values relating to work, leisure time, health, and relationships. Step 6 introduces cardiac-related fear, worry, and safety behaviors. Step 7 and 8 are optional modules that focus mainly on exposure training, but participants are encouraged to work through them, particularly participants identifying high levels of cardiac-related anxiety in step 6. Step 9 is the last step of the program and begins with a summary and overview of the treatment. This step encourages maintenance of what has been learned during the treatment and further development beyond the end of treatment. Each step also contains exercises for participants to work through. Where the exercise requires written content or homework tasks, participants are given feedback from the psychologist, accessed from the online portal, a feature supported by research into digital CBT as facilitating homework completion [40]. Screenshots of the digital intervention can be seen in Multimedia Appendices 2 and 3.

### Final Views of the Intervention

Findings were taken from the feedback provided at the final PRP meeting and based on the treatment version that the PRPs worked with up until that point.

#### Content

The final meeting with 4 members of the PRP group resulted in several small changes to the intervention. Participants expressed that it would be preferable if the heart-related fear steps, which were to be optional due to low activity in the testing, were instead mandatory additions to the first 5 steps, as the content was viewed as rather useful. With this in consideration, the first of the 3 cardiac-related fear steps was included as mandatory, while the 2 exposure steps were kept as optional. Implemented and attempted changes suggested by the PRPs in this meeting are presented later.

#### Platform Design

PRPs had some general agreements about the design features that should be included. They all agreed that the inclusion of pictures and images was necessary to make the intervention visually appealing and break up large blocks of text. They also welcomed the short video clips that featured certain scenarios that were used as examples during the treatment, and their positive feedback on the first clip even led to the inclusion of more clips throughout the treatment.

#### Terminology

**Figure 2:**
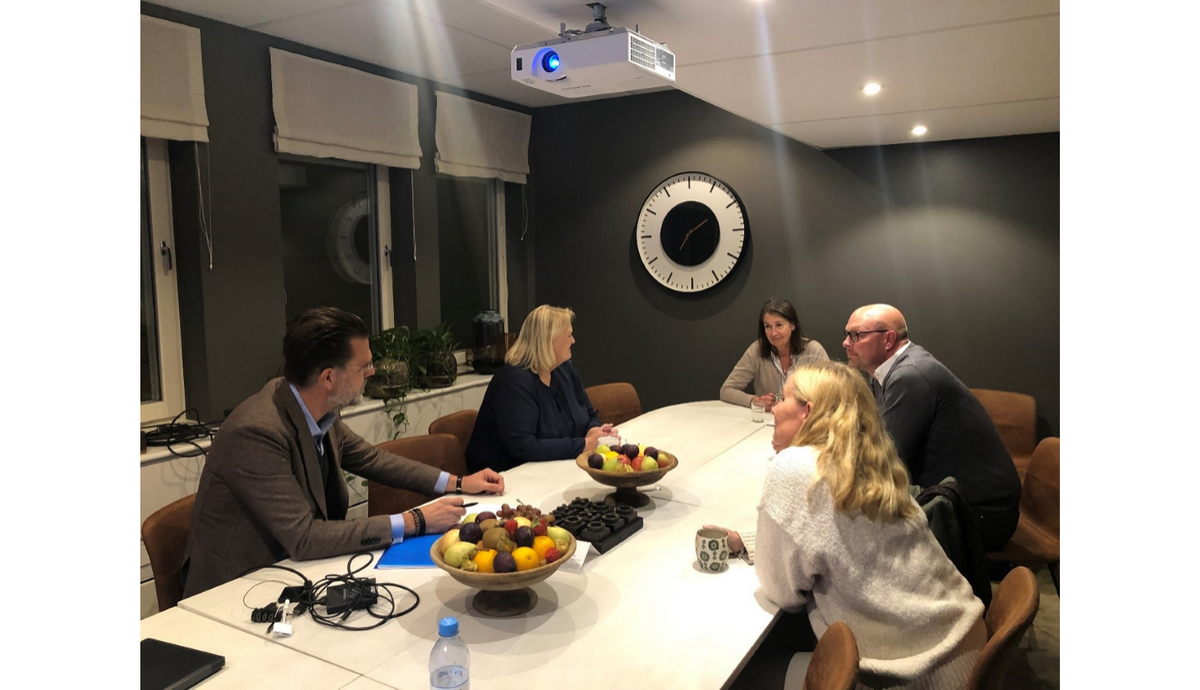
The patient research partners meeting during the final feedback concerning the internet-based cognitive behavioral therapy intervention.

PRPs expressed preference for the term MINOCA as opposed to something more general, such as “cardiac event,” but expressed the importance of explaining the term in detail at the start of treatment. This was especially important because the PRPs did not all fall under the same category of MINOCA; some were classified as having TTC, for example. By using the broader term MINOCA, it ensures that the examples given in the text and exercises are less likely to be misunderstood as irrelevant or exclusive to those unfamiliar with the term. Similarly, nonmedical terms that are commonly used (such as “broken heart syndrome” instead of takotsubo cardiomyopathy) were not considered appropriate by the panel, and they expressed discontent with their use in most contexts. Figure 2 shows the final PRP feedback meeting.

### Expert Panel’s View of the Intervention

Shortly after the final meeting with the PRPs, the expert panel was consulted for their views and advice on the intervention. Feedback revolved around the practical content, such as visual layout and design, as well as the treatment content. One suggestion from the expert panel was to include a visual aid to complement the progress of the participant as they work through the treatment steps. This was added to the intervention (Figure 1) and is changed with each additional step completed. Overall, the panel was enthusiastic and positive about the treatment program. Suggestions for change that were implemented upon receiving their feedback is also presented below (Table 2).

**Table 2:** Suggested changes made by patient research partners and the expert panel regarding the design and content of the intervention.

Group	Platform design		Treatment content	
	Identified suggestion	Modification	Identified suggestion	Modification
PRPs^a^	- Move the interview films - Include a chat function with the psychologist - Automatic message sent directly via email or SMS when something new is commented on in the portal	- Interview video content moved to introduction - Chat function considered but currently not implemented - Feature under construction, to be added	- Remove deadlines for homework tasks - Mandatory fear steps as opposed to only optional	- Replaced with recommended submission date instead - First cardiac-related fear step made mandatory, with 2 steps as optional
Expert panel	- Shorten interview video content - Add a follow-up video of patient interview - Visual representation of intervention progress - Captioning of videos	- Videos shortened - Video with patient with MINOCA^b^ added to include an update posttreatment - Stepwise diagram created and added to each step - Text captions added to videos	- Include measurements every step/week of the treatment - Add sleep-related content - Give a clearer message about exercise and physical exertion - Exposure therapy content that focuses less on habituation	- GAD-2^c^ and PHQ-2^d^ questionnaire measurements added weekly - Addition of “things to think about” regarding sleep in step 4 - Short text added to clarify “mixed messages” around the benefits of exercise for those who can - The heart-related fear section slightly rewritten to be more in line with an inhibitory learning model of exposure [41]

^a^PRPs: patient research partners.

^b^MINOCA: myocardial infarction with nonobstructive coronary arteries.

^c^GAD-2: 2-item Generalized Anxiety Disorder scale.

^d^PHQ-2: 2-item Patient Health Questionnaire.

### PRPs' Views Surrounding the Participatory Action Research Process

The final meeting with PRPs also included a short discussion about their participatory perspective of being involved in the development of an intervention. Many felt that being part of a panel contributing to the development of the intervention was a positive thing overall. They discussed that this process played a positive role in their lives and hopefully in the lives of other patients with MINOCA who will benefit from their input. Many expressed a motivation to contribute to better care for others, but also reported gaining some benefits to their own lives in being part of this process. Being surrounded by others with similar experiences was described by PRPs as something that led to a reduced feeling of loneliness, but they also reported that it exposed them to the fact that MINOCA affects others differently, something they saw as positive.

## Discussion

### Principal Results

The current paper describes the stepwise processes involved in the development of a web-based CBT program for patients with MINOCA experiencing psychological distress. Collaboration with PRPs and the expert panel resulted in a 6- to 9-week internet-delivered intervention with a CBT-focused approach aiming to reduce symptoms of high stress and anxiety. Continuous testing from PRPs, all of whom had received a diagnosis of MINOCA, helped to ensure that the intervention was relevant to the target patient group and hopefully more effective as a result.

Similar studies that have used PPI in intervention development have established beneficial impacts on the relevancy and acceptability of their interventions [42-44], and systemic reviews have also reported on the impact on study design and enhanced quality of research in general [45,46]. The outcome of our collaboration with 7 research partners enabled us to fulfill this aspect of PPI, hopefully producing an intervention better suited to the needs of the target user and that fulfilled the requirements shown in Textbox 1.

The process of using an iterative approach was the main advantage in the intervention creation. This meant that development was continuously modified so that each component was not finalized until participant feedback was provided. After feedback, it was fine-tuned even further, concluding with a final group meeting with the PRPs’ and the expert panel’s insight. This type of approach also ensured that the intervention was kept up to date during the development process, always tuned to the current needs or opinions of the patient group.

Participant feedback helped steer the direction of the development. While some treatment and design preferences might have appeared more in line with existing studies, the inclusion of a group of patients with a unique diagnosis brought some revelations to the surface that might not have otherwise been considered. A unique example of this is demonstrated in the use and consideration of certain terminology used to describe the diagnosis. PRPs expressed discontent with the term “broken heart syndrome” (often used, even among doctors), finding it to be downgrading or glamorizing the condition. A recent review of medical or nonmedical terms used to describe TTC supports this view and claimed that metaphorical language should be discouraged while the pathophysiological and diagnostic knowledge is still being established [47].

A couple of limitations should, however, be noted. First, the team worked with an existing health care platform to provide the intervention, and while many features were added in response to the PRP’s requests, it was not possible to cater to all requests, in part due to platform constraints. We could therefore only offer as much as the platform allowed. Some PRPs liked the idea of a video chat feature, for example. However, this feature was not possible to fully implement at the time of the feasibility testing phase, but it is on course to be available for later testing in a future randomized controlled trial.

Furthermore, while the work with PRPs ensured that patients with MINOCA were able to contribute their specific views and experiences towards the intervention, it should be noted that the transferability to current and future patients with MINOCA might be limited due to the sample being recruited from one main city region in Sweden. It should therefore be considered that across different hospitals in Sweden, the experience and views might differ with regard to treatment needs and rehabilitation. In addition, PRPs were included at different stages after the acute event, with one PRP having had MINOCA just a few weeks before inclusion, whereas another had a MINOCA 6 years prior. However, while the intervention will aim to be used just a few weeks after the MINOCA, putting the validity of the feedback from the PRPs with older MINOCA diagnoses into question, it is still felt to be valuable to get insight from PRPs who had MINOCA a long time ago.

Several challenges were inevitably experienced during the design process. As expected, the psychologists struggled at times to receive feedback that could be used to change the content more specifically. This mostly occurred when the PRPs did not feel like they related to that specific section of the intervention. However, since no group of patients is homogenous, it was not unexpected that the group would present with diverse problems and experiences. Scoring over a specific threshold on measures of stress, anxiety, or depression was not a requirement for the PRPs involved in the cocreation of the intervention, but it will be a prerequisite in the randomized control trial planned to follow. Therefore, some of the problems the intervention aimed to tackle might not have been personally problematic for the PRPs involved. Nevertheless, the PRPs agreed that the presented content and exercises under those circumstances were good to include and would be very helpful for the target user with MINOCA.

One future improvement feature to consider is individualization of the program for each participant. In the initial planning phases, we discussed the possibility of tailoring parts of the intervention, but this was deemed to result in a more complex intervention demanding too much of participants making informed choices and too difficult to construct. Since cardiac patients in previous internet-based interventions have described the treatment as burdensome and long [37,48], we aimed to produce a simple yet effective web-based treatment that would work for the target group and minimize dropout. However, individualized therapy in which participants can select the content they would like to work through themselves could be a future consideration for the mix of preferences patients with MINOCA might have with regard to psychological treatment. This was compensated for partly by offering the choice of the optional heart-related fear content, but future improvement could work towards making the intervention more customized. However, this intervention, as specified in the aforementioned a priori requirements (Textbox 1), aimed at offering treatment that can suit the majority of patients with MINOCA, something we believe was achieved. Therefore, customization of the intervention to a detailed degree could be something left to future studies.

### Conclusion

The process of involving PRPs in the development of an internet-based psychological treatment provided substantial insights and ideas. These contributed substantially to what we believe is a treatment both relevant and helpful for patients with MINOCA who also report moderate levels of stress, depression, or anxiety following their diagnosis. The diversity of the group enabled a range of viewpoints to be represented, and PRPs expressed that being part of the panel was a positive experience. Moreover, including an expert panel to provide feedback and suggestions for improvement ensured that input was considered from researchers and clinicians outside of the core research group. Using a design process that structuralized components of the intervention into prototypes and built upon these in an iterative manner with constant PRP feedback resulted in an intervention that has been tailored to specific MINOCA patient groups in focus. The next step of evaluation will be through a subsequent feasibility analysis and randomized controlled trial to test the result of this design process and the intervention’s effectiveness as a whole.

## Appendix

Multimedia Appendix 1 Translated interview guide from the final patient research partner meeting.

Multimedia Appendix 2 Screenshot of the intervention.

Multimedia Appendix 3 Screenshot of the intervention.

## Abbreviations

CAD: coronary artery disease
CBT: cognitive behavioral therapy
FGD: focus group discussion
iCBT: internet-based cognitive behavioral therapy
MI: myocardial infarction
MINOCA: myocardial infarction with nonobstructive coronary arteries
PPI: patient and public involvement
PRP: patient research partner
TTC: takotsubo cardiomyopathy
U-CARE: Uppsala University Psychosocial Care Programme
UCD: user-centered design
