# Supplementary material for: Designing a Web-Based Psychological Intervention for Patients With Myocardial Infarction With Nonobstructive Coronary Arteries: User-Centered Design Approach
Source: J Med Internet Res. 2020 Sep 17;22(9):e19066. doi: 10.2196/19066 (PMC7530693; doi:10.2196/19066)
Supplement: Multimedia Appendix 1 [file jmir_v22i9e19066_app1.docx]

About the Programme - Interview Guide (English translation)

**Inclusion and motivation**

1. What is important for participants to know before deciding to participate in the study or not, and thus the programme, from the beginning?
2. What has been important in order to continue being active in the programme?
3. What could increase motivation in order to be further active in the programme?
4. Has anything decreased your motivation?
5. Could something reasonably be able to completely destroy the motivation to participate in the programme?

**Structure**

1. Should all the material be worked with chronologically or is it good if certain material is available if needed, such as in a library (e.g. films and extra material)?
2. Would it be good to have clear deadlines between each step? If so, how should they be applied?
3. Regarding conversation with the psychologist:
4. Are phone calls, video calls and/or chat conversations preferred?
5. When in the program are they the most appropriate to have?
6. How often?
7. Should they be scheduled or is it okay to be called?
8. When in the day? Weekday?
9. Which focus is best for these calls?
10. Regarding reminders and prompts:
11. Automatic or telephone calls?
12. SMS and email?
13. With inactivity, when new questionnaires should be filled in and/or inactivity in the treatment?
14. Regarding written feedback on information from the psychologist:
15. What is good feedback?
16. How do you want to have the feedback?

**Contents**

1. What, if anything, do you generally like about the films in the program?
2. What (if anything) have the interview films added?
3. Should there be more? Longer? Shorter? With other interviewees?
4. What have the feature films (with examples) added (if anything)?
5. Should there be more? Longer? Shorter? Include other situations?
6. Have you recognised the examples given in the text?
7. Have they been good?
8. Should something change?
9. Tips and links, e.g. to 1177, is it valuable? Extra depth material?
10. The program has had different focuses. How does your situation fit:
11. Follow-up, maintenance, relapse prevention (the last part)
12. Stress
13. Behaviour change
14. Fear after a heart-related event

**Word Choices**

1. How do you look at the following word choices:
2. Steps / Weeks / Sections
3. Therapist / Psychologist / Support person
4. MINOCA / Heart event / Takotsubo
5. Fear / Anxiety / Concern
6. Library / Extras
7. How do you think the participant should be addressed in the program?
8. Direct: You? In third-person?
9. We?

**Technical and Layout**

1. How has everything worked in the technical sense for you?
2. What has worked less well?
3. What do you think about the look and design of the program?
