# Supplementary figures and images for: Designing a Web-Based Psychological Intervention for Patients With Myocardial Infarction With Nonobstructive Coronary Arteries: User-Centered Design Approach
Source: J Med Internet Res. 2020 Sep 17;22(9):e19066. doi: 10.2196/19066 (PMC7530693; doi:10.2196/19066)

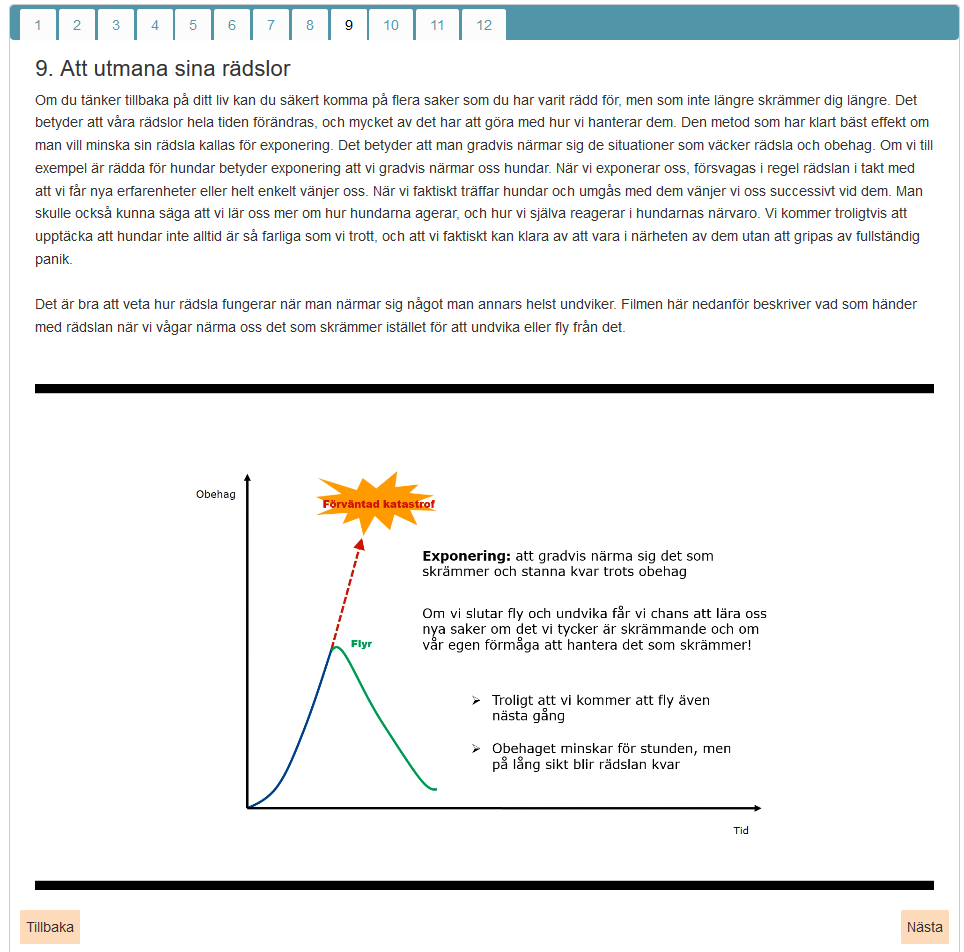

Supplement: Multimedia Appendix 2 [file jmir_v22i9e19066_app2.png]

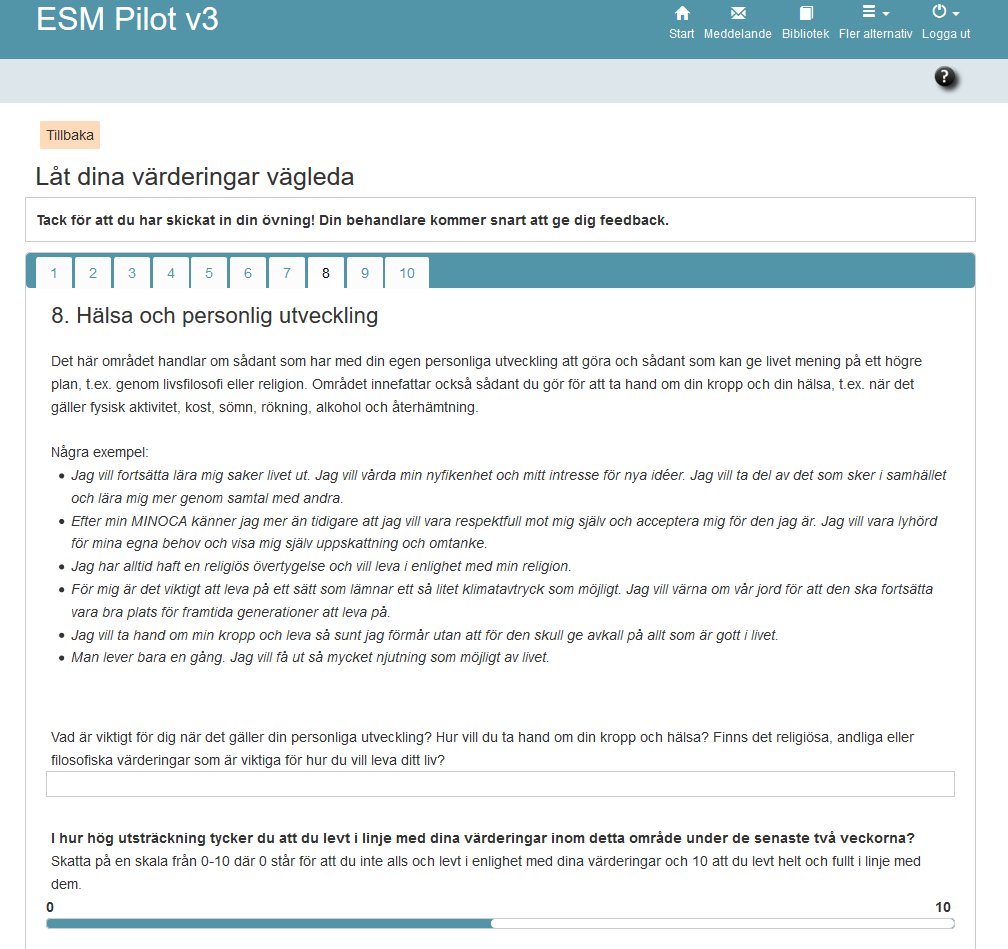

Supplement: Multimedia Appendix 3 [file jmir_v22i9e19066_app3.PNG]
